# Variability in Running Economy of Kenyan World-Class and European Amateur Male Runners with Advanced Footwear Running Technology: Experimental and Meta-analysis Results

**DOI:** 10.1007/s40279-023-01816-1

**Published:** 2023-03-02

**Authors:** Melanie Knopp, Borja Muñiz-Pardos, Henning Wackerhage, Martin Schönfelder, Fergus Guppy, Yannis Pitsiladis, Daniel Ruiz

**Affiliations:** 1 adidas Innovation, adidas AG, Herzogenaurach, Germany; 2 GENUD Research Group, Faculty of Health and Sport Sciences, University of Zaragoza, Saragossa, Spain; 3 Department of Sport and Health Sciences, Technical University of Munich, Munich, Germany; 4 Institute of Life and Earth Sciences, Heriot Watt University, Edinburgh, UK; 5 School of Sport and Health Sciences, University of Brighton, Eastbourne, UK

## Abstract

**Background:**

Advanced footwear technology improves average running economy compared with racing flats in sub-elite athletes. However, not all athletes benefit as performance changes vary from a 10% drawback to a 14% improvement. The main beneficiaries from such technologies, world-class athletes, have only been analyzed using race times.

**Objective:**

The aim of this study was to measure running economy on a laboratory treadmill in advanced footwear technology compared to a traditional racing flat in world-class Kenyan (mean half-marathon time: 59:30 min:s) versus European amateur runners.

**Methods:**

Seven world-class Kenyan and seven amateur European male runners completed a maximal oxygen uptake assessment and submaximal steady-state running economy trials in three different models of advanced footwear technology and a racing flat. To confirm our results and better understand the overall effect of new technology in running shoes, we conducted a systematic search and meta-analysis.

**Results:**

Laboratory results revealed large variability in both world-class Kenyan road runners, which ranged from a 11.3% drawback to a 11.4% benefit, and amateur Europeans, which ranged from a 9.7% benefit to a 1.1% drawback in running economy of advanced footwear technology compared to a flat. The post-hoc meta-analysis revealed an overall significant medium benefit of advanced footwear technology on running economy compared with traditional flats.

**Conclusions:**

Variability of advanced footwear technology performance appears in both world-class and amateur runners, suggesting further testing should examine such variability to ensure validity of results and explain the cause as a more personalized approach to shoe selection might be necessary for optimal benefit.

## Key Points

**Table:** 

Running economy of world-class Kenyan and amateur European runners with next-generation long-distance running shoes that contain advanced footwear technology varies greatly, with a range from a 11.4% benefit to a 11.3% detriment.
Meta-analysis results reveal an overall statistically significant medium benefit of advanced footwear technology on running economy when compared with traditional racing flats and confirmed the variability we report when examining the performance benefits of advanced footwear technology.
Our results suggest a more personalized approach to new footwear technology.

## Introduction

Kenyan elite runners win many international track and road distance races, which has stimulated research into the causes of this success [1–6]. When examining the geographical distribution of the top 20 running performances for male and female athletes in both middle- and long-distance events (800 m, 1500 m, 3000 m, 5000 m, 10,000 m, 5 km, 10 km, half-marathon, and marathon) in the past 5 years (since the last Olympic cycle: 5 August, 2016 to 29 August, 2021), 41.6% have been achieved by Kenyan athletes [7]. Such running performances depend on three main physiological factors: (1) an athletes’ maximal oxygen uptake (${\dot{\text{V}}}$O_2_max), (2) their fractional utilization of ${\dot{\text{V}}}$O_2_max or the ability of an athlete to sustain a high percentage of their ${\dot{\text{V}}}$O_2_max for long periods of time, and (3) their running economy [8–11]. Previous research examining the uniqueness specifically of Kenyan or other elite East African runners has suggested that of these, it is running economy that is particularly unique in this population [6, 10, 12]. Various studies have further attributed this especially to the anthropometric characteristics of East Africans with smaller body size, thinner lower legs, and a greater Achilles tendon moment arm with a shorter forefoot length [1, 10, 12–14].

**Fig. 1:**
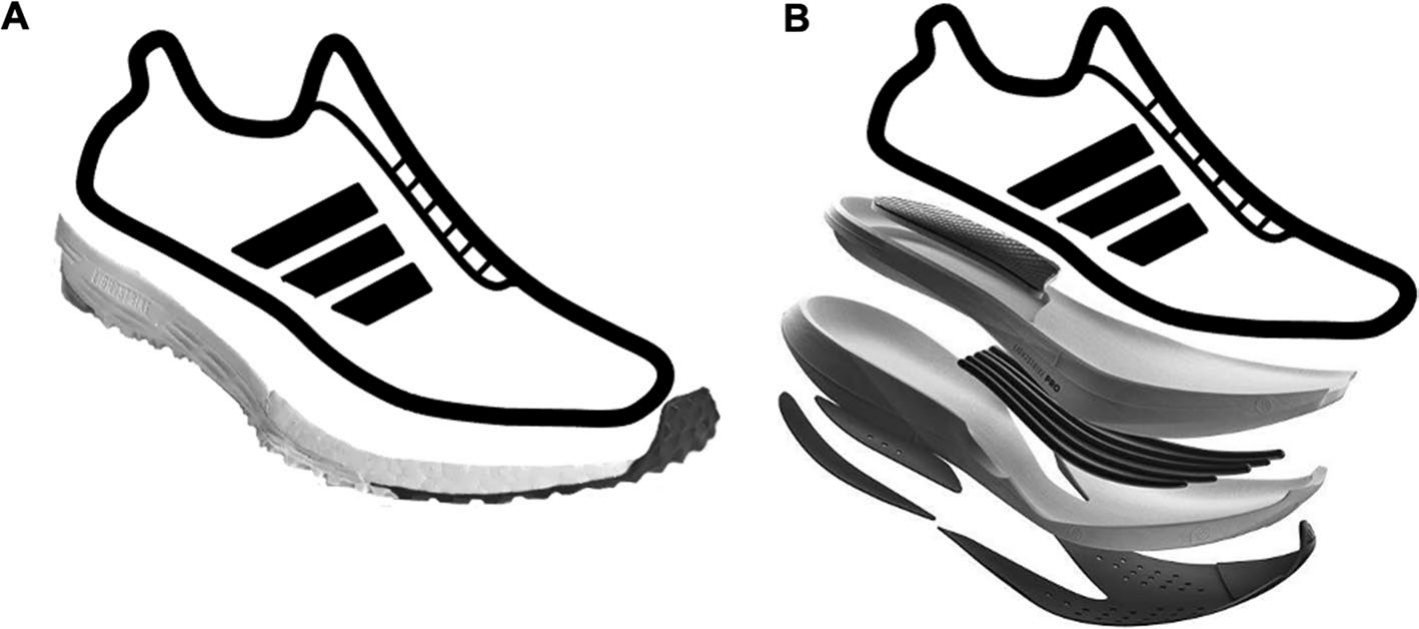
Schematic of different long-distance running shoes, including **A** a traditional racing flat, which is classically low to the floor with relatively thin soles with the focus here being to keep the shoes lightweight, and **B** advanced footwear technology, which consists of a curved stiff element in the forefoot of the shoe, as well as a high midsole stack height made up of a resilient, compliant, and lightweight foam

Running economy can be defined as the ability to move efficiently in terms of energy demand while running at a specified submaximal velocity and can be measured as the rate of oxygen uptake per kilogram body weight and minute (${\dot{\text{V}}}$O_2_ in mL O_2_/kg/min) at that speed [10, 11, 15, 16]. Previous work has reported that among elite runners with similar ${\dot{\text{V}}}$O_2_max levels, running economy can account for 65.4% of the variation observed in a 10-km race performance [17]. Running economy is affected by many factors including anthropometric, biomechanical, metabolic, neuromuscular, and cardiorespiratory efficiency [11]. One element that has gained interest in recent years is an athlete’s mechanical efficiency being affected by different footwear characteristics such as weight, cushioning, and longitudinal bending stiffness, all of which are included in recent technological advances in long-distance running shoes [18–21]. Previously published work has attributed the improvements of performance of such advanced footwear technology to various mechanisms [20, 22]. The advances in shoe technology themselves have been designed to maximize running economy while minimizing energy loss and consist of a curved stiff element component and a high midsole stack height made of a compliant, resilient, and lightweight foam (Fig. 1). The curved rigid element increases the longitudinal bending stiffness of the shoe and thereby creates a mechanism with a teeter-totter effect on the running mechanics, which occurs when a runner’s center of pressure overcomes the bending point of the curved structure and causes the reaction force to act on the heel perpendicular to the stiff element providing leverage during push-off [20, 23]. The high midsole stack height enhances this mechanism and allows for a more curved plate to be inserted into the midsole [20]. The compliant, resilient, lightweight foam material for the midsole ensures that the shoe weight remains light while still having a soft foam with a high-energy return as these have all been suggested to also affect performance [18–20].

The impact of advanced footwear technology on running events is reflected in the progression of world records, with every male and female world record starting from 5 km to the marathon broken by athletes wearing different versions of these shoes since their release [24]. Previous research completed on such footwear technology in the field quantifies this impact on performance, with data from the Strava fitness app on more than a million marathon and half-marathons revealing that shoes containing this new technology could improve race performance in sub-elite athletes, as individuals ran 4–5% faster in advanced footwear technology than runners wearing an average racing flat [25]. Similarly, Rodrigo-Carranza et al. showed that in a sub-cohort of top-100 men’s marathon performances from 2015 to 2019 that completed races in both advanced footwear technology and traditional flats, 29 of 40 athletes (72.50%) improved their performance with this type of footwear [26]. This is also supported by various laboratory-based running economy studies comparing advanced footwear technology to traditional racing flats in sub-elite athletes, suggesting that the design of these shoes reduces the energy cost of running on average by about 2.7–4.4%, thereby benefiting overall running performance [15, 27–30].

While previous studies have compared the running economy of non-elite runners wearing different shoe technologies in relatively controlled laboratory settings [15, 27–30], no study has examined the variability in running economy of the main beneficiaries (i.e., world-class athletes). Knowing this, the primary aim of this study was to answer the research question: how does the variability in physiological response in terms of running economy on a laboratory treadmill in advanced footwear technology compare to a traditional racing flat in world-class Kenyan distance runners (half-marathon mean time: 59:30 min:s) versus European amateur runners? Based on the obtained results, we decided to systematically search the literature for similar relevant studies and conducted a post-hoc meta-analysis to confirm the found range of variability, and better understand the overall effect of advanced footwear technology.

## Materials and Methods

### Participants

Fifteen subjects volunteered to participate in this study and were classified as either world class or amateur. Runners with current or recent injuries that prevented them from training were excluded, as well as those uncomfortable with running on a treadmill. Shoe size was also part of the inclusion criteria because of shoe cost considerations. One participant dropped out as he struggled to run on a treadmill, meaning 14 participants were finally included for analysis in this study.

The world-class cohort comprised seven male world-class Kenyan runners (mean ± standard deviation, age: 22.7 ± 3.2 years, height: 1.7 ± 0.05 m, mass: 59.9 ± 4.8 kg, body mass index: 19.7 ± 0.6 kg/m^2^, ${\dot{\text{V}}}$O_2_peak: 75.9 ± 3.5 mL/kg/min) (Table 1) [31]. These runners were recruited through sponsorship deals with collaborating companies and were all professional road racing athletes who had an official mean personal record for the half-marathon of 59:30 ± 0:48 min:s, and a 10-km personal best of 27:33 ± 0:41 min:s. The amateur cohort consisted of seven well-trained male amateur European runners, who at the time of measurement were training daily, (mean ± standard deviation, age: 28.1 ± 4.2 years, height: 1.8 ± 0.03 m, mass: 72.1 ± 7.0 kg, body mass index: 21.9 ± 1.8 kg/m^2^, ${\dot{\text{V}}}$O_2_peak: 62.3 ± 5.1 mL/kg/min) and volunteered to take part in this research (Table 1). All participants gave written informed consent to being a part of this study after they understood the experimental procedures, potential injury risks, and possible benefits.

**Table 1:** Participant descriptive and physiological characteristics for each of the measured cohorts

Variable	World class	Amateur	*p*-value
	*n* = 7	*n* = 7	
Age (years)	22.7 ± 3.2	28.1 ± 4.2	0.020*
Height (cm)	174.3 ± 4.9	181.4 ± 2.6	0.008*
Weight (kg)	59.9 ± 4.8	72.1 ± 7.0	0.003*
${\dot{\text{V}}}$O_2_peak (mL/kg/min)	75.9 ± 3.5	62.3 ± 5.1	< 0.001*
${\dot{\text{V}}}$O_2_peak (L/min)	4.53 ± 0.43	4.49 ± 0.48	0.870
v${\dot{\text{V}}}$O_2_peak (km/h)	22.3 ± 0.6	18.8 ± 1.2	< 0.001*

Data shown are mean ± standard deviation

$\dot{V}$*O*_*2*_*peak* maximal oxygen uptake, *v*$\dot{V}$*O*_*2*_*peak* velocity at $\dot{V}$*O*_*2*_*peak*, Student’s *t* test

*Significance (*p* < 0.05)

### Shoes

Throughout the experimental protocol, analyzed shoe conditions included a commercially available traditional racing shoe (FLAT) used by the subjects regularly for their own training, as well as three different commercially available models of AdvFootTech (1–3) that differed in their geometry and weight (Table 2). As all athletes were the same shoe size, everyone tested in UK 8.5 (US 9/EU 42 2/3).

**Table 2:** Descriptive characteristics of the AdvFootTech and FLAT

Shoe label	Mass (g)	Forefoot stack height (mm)	Rearfoot stack height (mm)	Heel-to-toe drop (mm)	Energy return (%)	Stiff element?
AdvFootTech 1	225	31.5	39	8.5	High	Yes
AdvFootTech 2	210	29.5	39.5	10	High	Yes
AdvFootTech 3	196	31	39.5	8.5	High	Yes
FLAT	197	19	24	5	Low	No

NShoe characteristics based on size UK 8.5/US 9

Energy return classification: low: < 70%; medium: 70–80%; high: > 80%

*AdvFootTech* advanced footwear technology, *FLAT* traditional racing flat

### Experimental Protocol

**Fig. 2:**
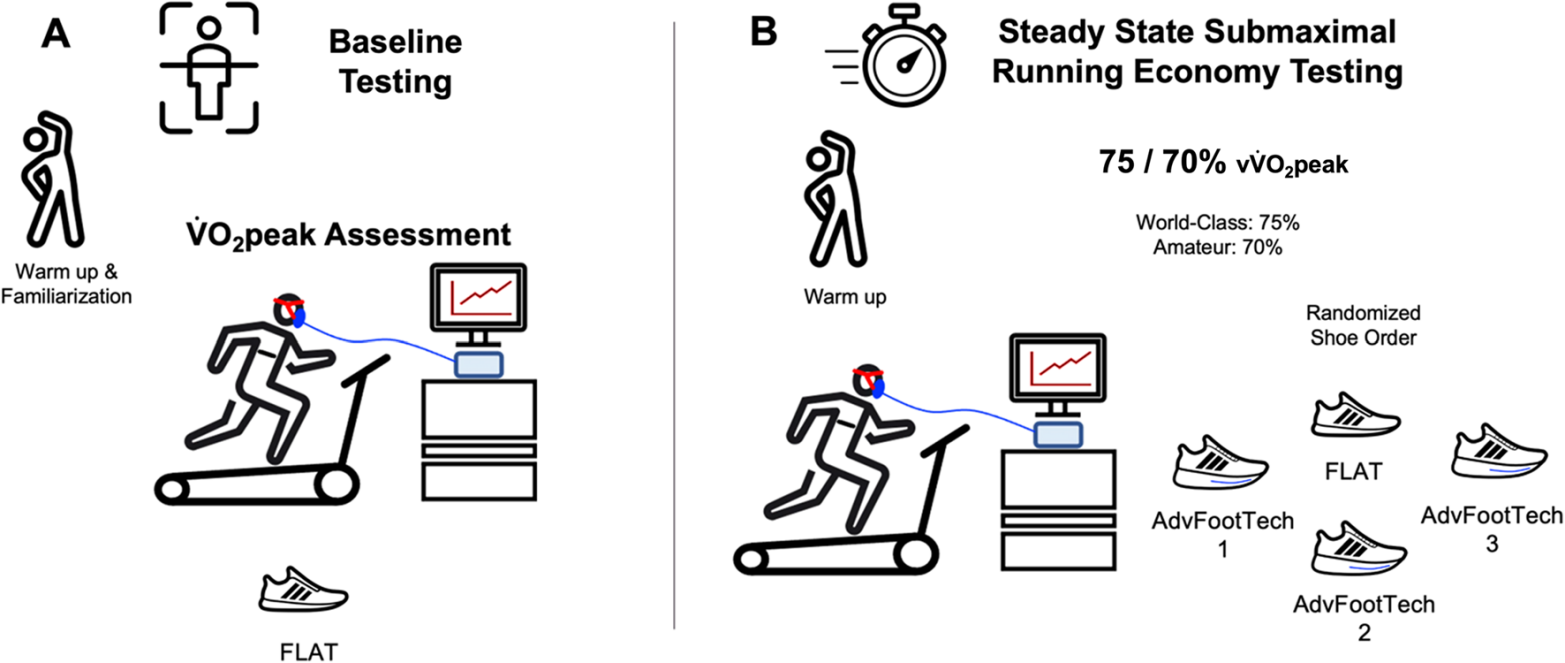
Illustration of the methods protocol of the present study. **A** For visit 1, we collected baseline information of the subjects, which included conducting a maximal oxygen uptake (${\dot{\text{V}}}$O_2_peak) assessment. **B** On the second day of testing, we then assessed the running economy of both traditional racing flat (FLAT) and different advanced footwear technology (AdvFootTech) models. *v*$\dot{V}$*O*_*2*_*peak* velocity at $\dot{V}$*O*_*2*_*peak*

This study comprised two laboratory visits occurring on separate days, with a 24-h pause for recovery, at the adidas Sports Science Research Laboratory in Herzogenaurach, Germany located close to sea level at an altitude of 300 m (Fig. 2). During the first session, we collected ${\dot{\text{V}}}$O_2_peak and baseline measurements. In the subsequent session, we measured running economy in different footwear conditions at either 75% (world class) or 70% (amateur) of the corresponding velocity to the measured ${\dot{\text{V}}}$O_2_peak, (v${\dot{\text{V}}}$O_2_peak) [32]. We chose the 75/70% of v${\dot{\text{V}}}$O_2_peak as this was a submaximal speed related to speeds these subjects would use when running at a marathon pace.

To ensure consistency and avoid any confounding effects of circadian rhythm [33], we tested participants at the same time of day and encouraged them to match their diet, sleep, and training patterns prior to each session. Furthermore, to ensure the athletes felt comfortable being in a foreign environment and understood all that was asked of them, their coach as well as manager traveled with them and helped with testing. This favored a clearer communication between the research team and the athletes.

#### Visit 1

In this preliminary visit, we collected physiological baseline and anthropometric measurements. Throughout the whole experiment, all treadmill sessions were conducted in the same standardized laboratory chamber (mean ± standard deviation, temperature: 25.5 ± 1.1 °C, humidity: 60.2 ± 8.8%, pressure: 980.7 ± 4.9 mBar) on a HP Cosmos motorized treadmill (Venus 200/75; h/p/cosmos sports and medical GmbH, Nussdorf-Traunstein, Germany) set at a 1% gradient to mimic the energetic cost of running outdoors [34]. Given that some runners were not accustomed to treadmill running or using a ${\dot{\text{V}}}$O_2_peak protocol, we familiarized subjects during a 15-min session on the treadmill with increasing speeds. Once they felt comfortable running on a treadmill, we fitted each athlete with a heart rate monitor (Polar H7; Polar Electro Oy, Kempele, Finland) and face mask (7450 Series V2 Mask; Hans Rudolph, Inc., Shawnee, KS, USA), connected to the MetaMax 3B portable cardiopulmonary gas exchange measuring device (CORTEX Biophysik GmbH, Leipzig, Germany). We then collected respiratory parameters from the subjects using an automated breath-by-breath method, via the measurement and evaluation software, MetaSoft Studio (CORTEX Biophysik GmbH, Leipzig, Germany). Before each testing session, we calibrated this system according to the manufacturer’s instructions [35, 36].

To assess maximal aerobic capacity, athletes completed a ${\dot{\text{V}}}$O_2_peak ramp test using an incremental speed protocol with a continuous 1% incline. For this, athletes ran in the new pairs of the traditional racing FLAT test condition. For the world-class athletes, this test started at 10 km/h for 2 min and increased progressively at 1 km/h/min until volitional exhaustion. Amateurs completed the same protocol starting at 8 km/h. During this test, we verbally encouraged all athletes to ensure a maximal output was reached.

Upon completion, two experienced exercise physiologists detected and agreed upon ventilatory thresholds and ${\dot{\text{V}}}$O_2_peak values. For all cardiorespiratory data, we cleaned the breath-by-breath raw data by removing outlying data points that were more than two standard deviations away from the mean of a seven-breath window. After these outliers were removed, data were smoothed further by taking a moving seven-breath average. The ${\dot{\text{V}}}$O_2_max value was recorded as the highest cleaned and smoothed value during the test. As we did not repeat a verification test to confirm these values, the highest recorded ${\dot{\text{V}}}$O_2_ value will be defined as a ‘${\dot{\text{V}}}$O_2_peak’ [37]. The measured v${\dot{\text{V}}}$O_2_peak (km/h) was also recorded and used to prescribe the running speed for the running economy tests during visit 2. Ventilatory threshold data as well as previously recorded personal bests of each athlete were used to ensure the selected speeds were sufficient in obtaining testing data that are relevant to racing and would not be affected by fatigue.

#### Visit 2

During visit 2, we assessed running economy for each of the different shoes at 75% of v${\dot{\text{V}}}$O_2_peak (17.0 ± 0.4 km/h) for world-class athletes and 70% (13.1 ± 1.0 km/h) for amateur athletes. When subjects arrived, they first completed a 6-min standardized warm-up in the FLAT. This was then followed by a 12-min break during which we prepared the equipment for the test that consisted of 6-min bouts with a 12-min rest between bouts. Before each new treadmill trial, athletes changed their shoes for the next bout. The last 30 s of this break were recorded on the treadmill to obtain resting values.

From the recorded measurements, we calculated running economy, oxygen cost of transport, and energetic cost using the Péronnet and Masicotte equation expressed in mL/kg/min, mL/kg/km, and W/kg, respectively, from the ${\dot{\text{V}}}$O_2_ data during the 60-s period from minute 4 to 5 of each test [38].

### Data and Statistical Analysis

All data analysis and statistical tests were performed using RStudio [39]. Statistical analyses of the data were performed using the R package ‘stats’ (version 4.0.0) in RStudio [39, 40] using the traditional level of significance (*p* < 0.05). Power and sample size calculations were performed using the R package ‘pwr’ (version 1.3-0) in RStudio also using the traditional level of significance (*p* < 0.05), 80% power, and four different groups for the four different shoes. We conducted a Student’s *t* test on the descriptive characteristics to analyze population differences between the measured world-class and amateur athletes. Additionally, an analysis of variance test with repeated measures and a Bonferroni post-hoc correction were conducted on the steady-state physiological data [41, 42].

### Systematic Review and Meta-analysis

To confirm the found range of variability with the previously published literature, and better understand the overall effect of advanced footwear technology, we conducted a systematic electronic search of relevant studies and a related meta-analysis.

 For this retrospective systematic literature search, Scopus, SPORT-Discus, PubMed, Web of Science, and Footwear Science databases were searched using the terms “Racing Shoes” and “Running Shoes + Running Economy” through 21 November, 2021. Inclusion criteria for this review were studies that (1) examined the running performance effect of different versions of advanced footwear technology for road running compared to a traditional racing flat control condition; and (2) measured the running economy (mL/kg/min) of this comparison. Additional secondary outcome measures including oxygen cost of transport (mL/kg/km) and energetic cost (W/kg) were also analyzed to provide a bigger picture of the effects of such new technology on running performance. These results were then pooled using Hedge’s *g* for a standardized effect size [43] and the inverse heterogeneity (IVhet) model using the Epigear Meta XL software (version 5.3) [44]. We further analyzed outcomes of the meta-analysis using a *z*-score for significance, Cochran’s *Q* statistic for heterogeneity, and I-squared for inconsistency [45] and assessed the risk of bias using the Cochrane Risk of Bias Instrument for RCTs (RoB 2) [46].

## Results

### Running Economy

**Fig. 3:**
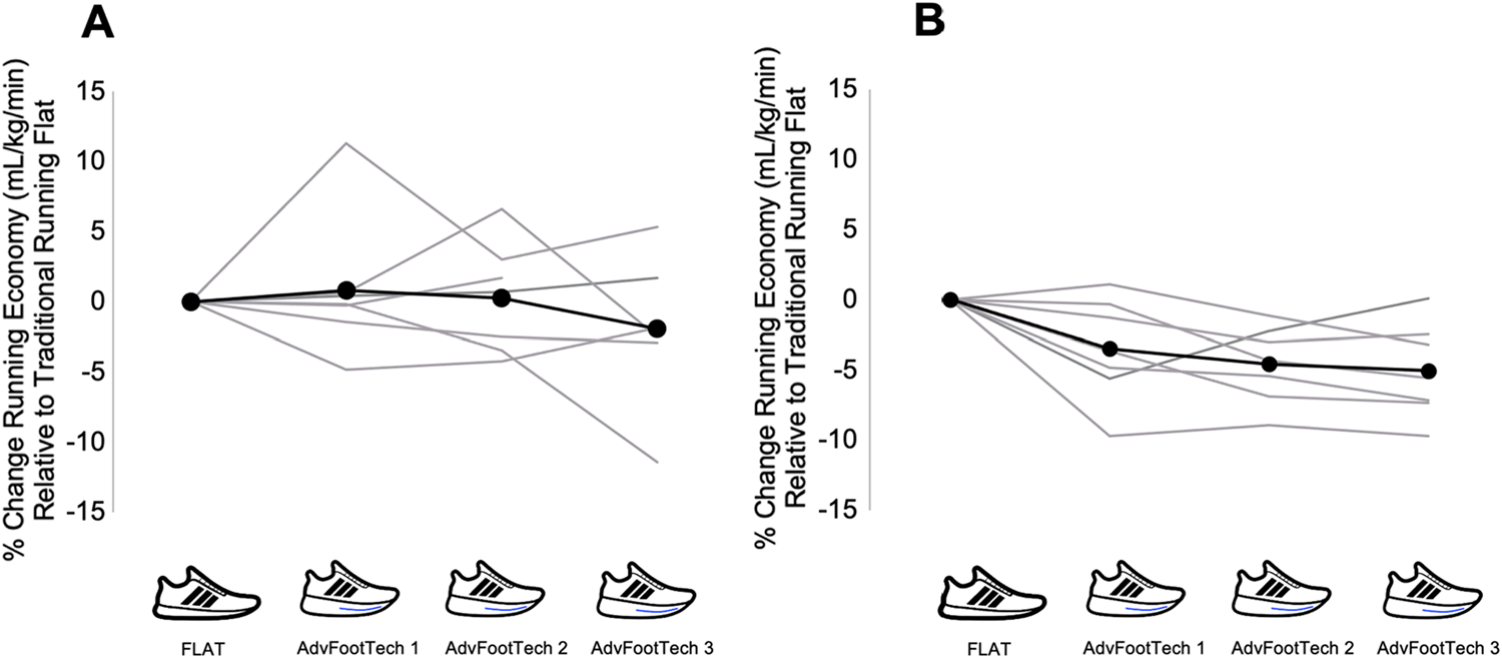
Percentage change in steady-state running economy oxygen consumption (mL/kg/min) relative to a traditional running flat (FLAT) in different shoe conditions for both **A** world-class and **B** amateur populations. These shoes include a FLAT on the far left as well as three different advanced footwear technology (AdvFootTech) conditions. Here, a negative percentage change indicates less oxygen consumption at a given speed and therefore a better running economy

From the available dataset (*n* = 14), for running economy there was a significant difference between shoe types in the amateur athletes (*F*(3) = 8.308, *p* = 0.001) where running economy in the advanced footwear technology was significantly lower than in the FLAT. Compared to the FLAT shoe, amateur athletes saw running economy improved by 3.5 ± 3.7% (*p*_Bonferroni_ = 0.042) with AdvFootTech 1, 4.6 ± 2.7% (*p*_Bonferroni_ = 0.005) with AdvFootTech 2, and 5.0 ± 3.4% (*p*_Bonferroni_ = 0.002) with AdvFootTech 3 (Fig. 3B, Table 3), with no significant differences between the three advanced footwear technology conditions.

**Table 3:** Steady-state physiological results for each of the different AdvFootTech and FLAT models separated between the world-class and amateur cohorts as well as statistical findings of the whole combined sample

Variable	World class (mean ± SD)				Among world-class subjects	Amateur (mean ± SD)				Among amateur subjects	Combined sample		
	*n* = 7					*n* = 7					Main effect shoes within subjects	Main population effect between subjects	Interaction effect within subjects
	FLAT	AdvFootTech 1	AdvFootTech 2	AdvFootTech 3	Repeated-measures ANOVA	FLAT	AdvFootTech 1	AdvFootTech 2	AdvFootTech 3	Repeated- measures ANOVA			
Running economy (mL O_2_/kg/min)	54.5 ± 2.0	54.9 ± 1.6	54.7 ± 2.8	53.5 ± 3.1	*F* = 0.743 *p* = 0.541	47.7 ± 2.6	46.1 ± 3.2^†^ *p*_Bonf_ = 0.043	45.5 ± 2.1^†^ *p*_Bonf_ = 0.004	45.3 ± 1.9^†^ *p*_Bonf_ = 0.002	*F* = 8.308 *p* = 0.001*	*F* = 3.360 *p* = 0.030*	*F* = 46.608 *p* < 0.001*	*F* = 1.741 *p* = 0.177
Oxygen cost of transport (mL O_2_/kg/km)	192.3 ± 8.1	193.8 ± 6.6	192.9 ± 11.8	188.7 ± 9.1	*F* = 0.875 *p* = 0.474	220.2 ± 12.3	212.3 ± 5.0^†^ *p*_Bonf_ = 0.047	209.9 ± 8.8^†^ *p*_Bonf_ = 0.006	208.9 ± 10.4^†^ *p*_Bonf_ = 0.003	*F* = 7.511 *p* = 0.002*	*F* = 4.245 *p* = 0.012*	*F* = 20.757 *p* < 0.001*	*F* = 2.478 *p* = 0.077
Energetic cost (W/kg)	19.4 ± 0.7	19.6 ± 0.6	19.5 ± 1.0	19.0 ± 1.3	*F* = 0.836 *p* = 0.493	16.9 ± 0.9	16.2 ± 1.2^†^ *p*_Bonf_ = 0.018	16.0 ± 0.8^†^ *p*_Bonf_ = 0.002	15.9 ± 0.7^†^ *p*_Bonf_ = < 0.001	*F* = 10.007 *p* < .001*	*F* = 3.572 *p* = 0.024*	*F* = 47.887 *p* < 0.001*	*F* = 1.886 *p* = 0.150
Respiratory exchange ratio	0.92 ± 0.02	0.93 ± 0.02	0.93 ± 0.02	0.90 ± 0.05	*F* = 1.001 *p* = 0.416	0.91 ± 0.03	0.88 ± 0.02^†^ *p*_Bonf_ = 0.029	0.88 ± 0.03^†^ *p*_Bonf_ = 0.016	0.88 ± 0.03^†^ *p*_Bonf_ = 0.005	*F* = 6.518 *p* = 0.004*	*F* = 2.741 *p* = 0.058	*F* = 4.935 *p* = 0.048*	*F* = 1.663 *p* = 0.193
Heart rate (bpm)	158.4 ± 8.8	157.7 ± 8.5	157.3 ± 10.1	155.6 ± 11.2	*F* = 0.919 *p* = 0.453	160.3 ± 5.9	157.2 ± 7.2	160.1 ± 6.5	158.8 ± 7.5	*F* = 1.527 *p* = 0.242	*F* = 1.542 *p* = 0.221	*F* = 0.278 *p* = 0.609	*F* = 1.072 *p* = 0.373
% Change in running economy to traditional running FLAT	0.0 ± 0.0	0.8 ± 5.0	0.3 ± 3.9	− 1.9 ± 5.6	*F* = 0.74 *p* = 0.543	0.0 ± 0.0	− 3.5 ± 3.7^†^ *p*_Bonf_ = 0.042	− 4.6 ± 2.7^†^ *p*_Bonf_ = 0.005	− 5.0 ± 3.4^†^ *p*_Bonf_ = 0.002	*F* = 7.969 *p* = 0.001*	*F* = 3.579 *p* = 0.023*	*F* = 4.170 *p* = 0.066	*F* = 2.039 *p* = 0.126

*AdvFootTech* advanced footwear technology, *ANOVA* analysis of variance, *FLAT* traditional racing flat, *SD* standard deviation

*Significant difference (*p* < 0.05)

^†^Shoes with value significantly different to the FLAT

Both the world-class and amateur athletes showed a large inter-individual variability with individual trials showing a ± 11.4% variation in performance (Fig. 3). When examining the individual advanced footwear technology conditions for the world-class population, the inter-individual range in overall performance changes of all included subjects vary by 14.6% on average for the different shoes. A similar pattern is also seen in the amateur population where values here range from a 9.7% benefit to a 1.1% drawback for advanced footwear technology when compared to the flat for a narrower inter-individual total range of 10.8% (Fig. 3B). For this population, the individual advanced footwear technology range in performance changes was narrower than that of the world-class population for an average of a 9.5% difference between the maximum and minimum percent change per shoe. Via a time and running economy interaction analysis, we ensured the shoe order did not have a significant effect on the described results (world-class: *p* = 0.61; amateur: *p* = 0.67).

In Table 3, we present the results for running economy, oxygen consumption, and percentage change in running economy in the advanced footwear technology models compared to a traditional running flat for both the world-class and amateur cohorts. Here, we compare the different shoes among cohorts, stratifying the data according to the amateur or world-class athlete results, as well as global effects comparing all tested subjects.

### Systematic Review Study Characteristics

**Fig. 4:**
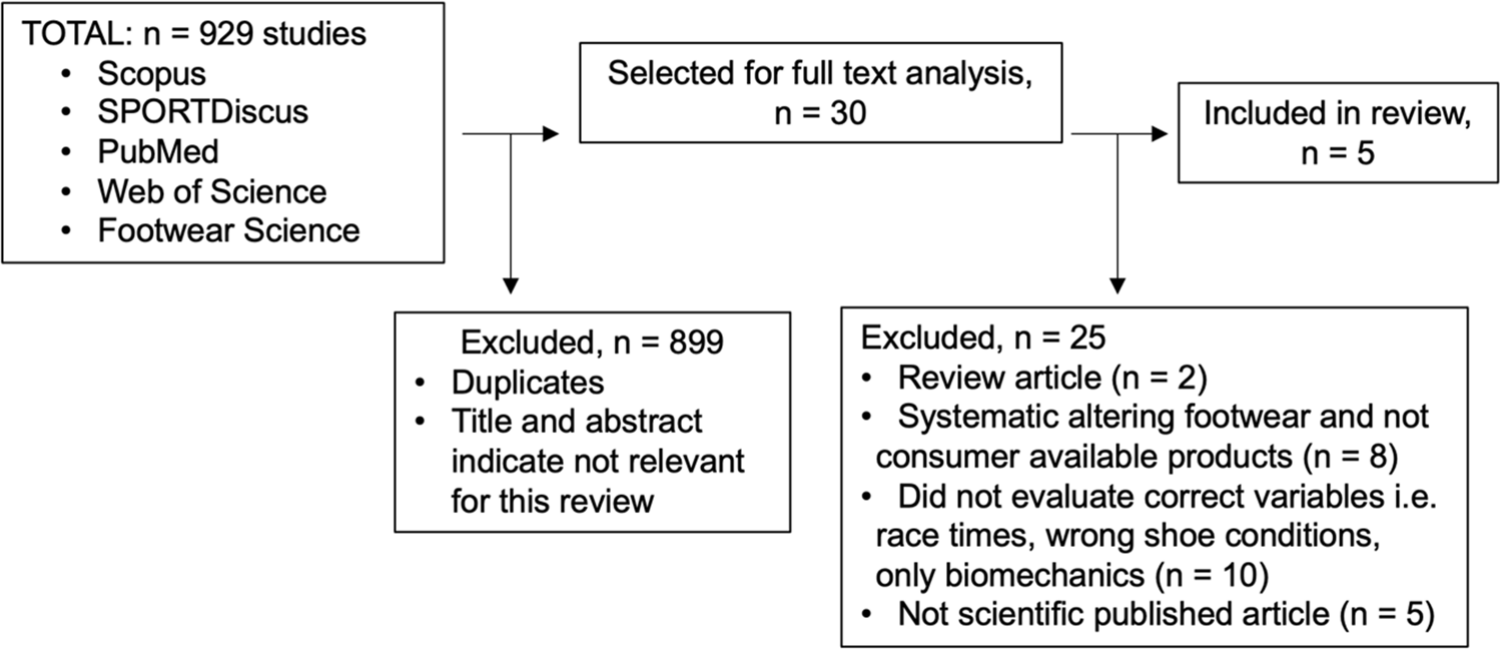
Flow chart showing study selection. Adapted from the PRISMA flow diagram [60]

From the initial search that resulted in 929 studies, 30 were selected for a full-text analysis after excluding by duplicates, title, and abstract, and five studies were finally included after fulfilling the inclusion criteria (Fig. 4). All examined studies were randomized crossover trials investigating a range of recreational to highly trained runners with a combined average measured ${\dot{\text{V}}}$O_2_peak of 67.1 ± 8.2 mL/kg/min. All studies examined a steady-state running analysis on a treadmill with different advanced footwear technology shoes compared to traditional racing flats, with Hébert-Losier et al. also including participants’ own shoes and spray painting the others to blind participants to model details [27]. Of the five studies, Barnes and Kilding was the only experiment to also include a female cohort [15]. Examined footwear conditions of the studies included in the meta-analysis are described in Table 4, please note data of shoe conditions irrelevant for this study, such as track spikes, were excluded in the meta-analysis [15]. When repeated conditions were used for the meta-analysis comparison, the corresponding conditions were divided by the number of repeated comparisons to ensure no double counting of effects. The testing was conducted at a variety of different speeds either between 14 and 18 km/h or in the case of Hébert-Losier et al., at different speeds relative to ${\dot{\text{V}}}$O_2_peak [27]. Hereby, we decided to subgroup the analysis based on the speed at which physiological variables were measured according to the protocols. We included four different speed categorizations starting with a very low speed that included 60% of v${\dot{\text{V}}}$O_2_peak where the speed was 11.0 ± 0.6 km/h; the low speed category included those conditions measured at 14 km/h for both men and women or 70% of v${\dot{\text{V}}}$O_2_peak with a speed of 12.9 ± 0.7 km/h; the medium-speed category included 16 km/h for men, 15 km/h for women, and 80% of v${\dot{\text{V}}}$O_2_peak with a speed of 14.7 ± 0.8 km/h; finally, the high-speed category included 18 km/h for men, and 16 km/h for women.

**Table 4:** Descriptive characteristics of shoe products included in the meta-analysis

Shoe label	Mass (g)	Forefoot stack height (mm)	Rearfoot stack height (mm)	Heel-to-toe drop (mm)	Midsole material	Stiff element?
AdvFootTech 1	225	31.5	39	8.5	n/a	Yes
AdvFootTech 2	210	29.5	39.5	10	n/a	Yes
AdvFootTech 3	196	31	39.5	8.5	n/a	Yes
AdvFootTech 4 [15, 27–29]	195	21	31	10	PEBA	Yes
AdvFootTech 5 [30]	196	32	40	8	PEBA	Yes
AdvFootTech 6 [30]	210	27	35	8	n/a	Yes
AdvFootTech 7 [30]	207	24	34	10	TPU	Yes
AdvFootTech 8 [30]	213	30	35	5	EVA	Yes
AdvFootTech 9 [30]	207	33	38	5	n/a	Yes
AdvFootTech 10 [30]	213	31	39	8	PEBA	Yes
AdvFootTech 11 [30]	210	36	40	4	PEBA	Yes
FLAT	197	19	24	5	TPU	No
FLAT 2 [28, 29]	181	15	23	8	EVA	No
FLAT 3 [28]	221	13	23	10	TPU	No
FLAT 4 [15, 29]	224	13	23	10	TPU	No
FLAT 5 [27]	130	13	13	1	TPU	No
FLAT 6 [27]	313 ± 44	n/a	26.0 ± 7.9	9.4 ± 6.7	Varies	No
FLAT 7 [30]	210	21	30	9	EVA	No

Shoe characteristics based on size UK 8.5/US 9 and obtained from original journal articles used in the meta-analysis or measurements conducted from RunningWarehouse.com. FLAT 6 varies (mean ± standard deviation) as it is a combination of the participants own footwear and includes sizes varying from US 8.5 to 12. Missing information (n/a) is because of the confidentiality of midsole material or missing information in the examined studies

*AdvFootTech* advanced footwear technology, *EVA* ethylene–vinyl acetate, *FLAT* traditional racing flat, *n/a* not available, *PEBA* polyether block amide, *TPU* thermoplastic polyurethane

Considering the risk of bias assessment of the included studies, all studies had some concerns for the category of bias arising from period and carryover effects, given the unknown effect of the physiological starting point between the trials and what carryover or how long a carryover might be with regard to running in advanced footwear technology. The overall risk of bias across all studies was of some concern owing to the similarities in the protocol of the study and the period and carryover effects.

### Meta-analysis Primary Outcome Measure: Running Economy

**Fig. 5:**
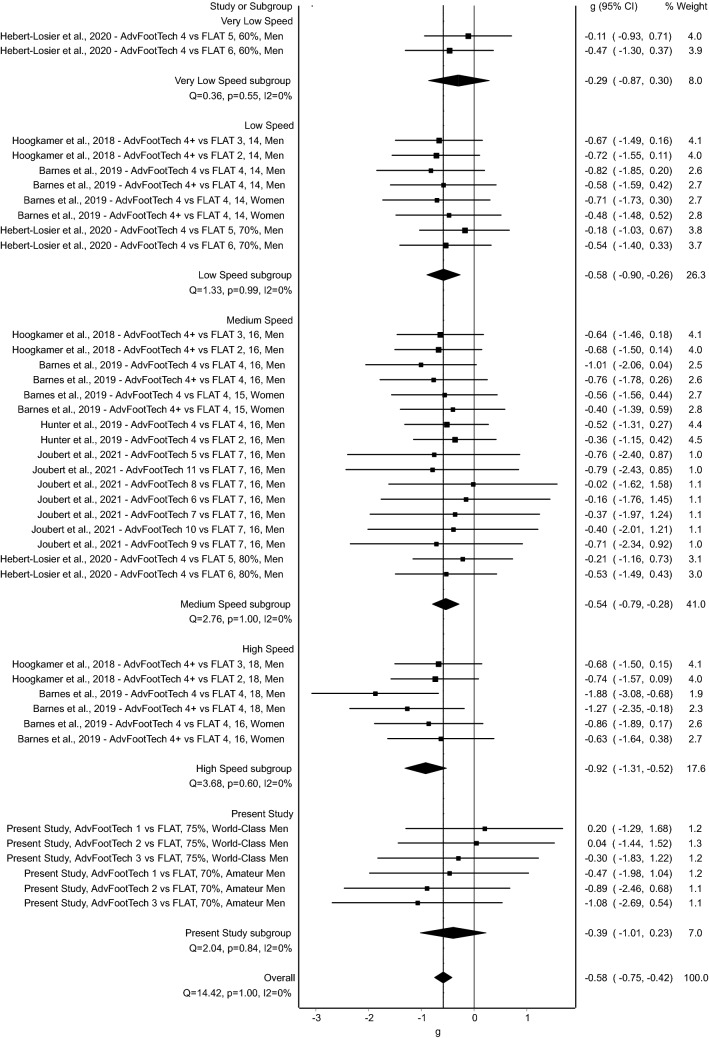
Forest plot displaying running economy (mL/kg/min) comparisons between advanced footwear technology (AdvFootTech) and traditional racing flats (FLAT) sub-categorized into different speeds. Study labels consist of the study name, the examined AdvFootTech versus FLAT condition where + indicates conditions that are weight matched, the speed either in km/h or as a % of peak, and the examined population. *CI* confidence interval

The meta-analysis of running economy (mL/kg/min) in all five examined studies comparing different advanced footwear technology to racing flat conditions revealed a statistically significant benefit of advanced footwear technology on running economy measures with an overall medium effect of − 0.58 [mean (95% confidence interval); *g* = − 0.58 (− 0.75, − 0.42), *Z* = − 6.86 (*p* < 0.001)], where a negative value indicates improved efficiency when running (Fig. 5). When sub-grouped by speed, the analysis showed a small effect [*g* = − 0.29 (− 0.87, 0.31)] at very low speeds, a medium effect [*g* = − 0.58 (− 0.90, − 0.26)] at low speeds, a medium effect [*g* = − 0.54 (− 0.79, − 0.28)] at medium speeds, and a large effect [*g* = − 0.92 (− 1.31, − 0.52)] at high speeds. Incorporating the data presented in this study, results are showing an overall medium effect [*g* = − 0.39 (− 1.01, 0.23)]. When this sub-analysis is further distributed by population, the world-class subgroup showed a small effect [*g* = − 0.02 (− 0.88, 0.85)], and the amateur subgroup showed a large effect [*g* = − 0.80 (− 1.70, 0.10)]. In this analysis, no statistically significant heterogeneity, as assessed via *Q*, was found (*Q* = 14.42, *p* = 1.00) and inconsistency, as assessed using *I*^2^ as an extension of *Q*, was very low (*I*^2^ = 0%) [45].

### Meta-analysis Secondary Outcome Measures: Oxygen Cost of Transport and Energetic Cost

**Fig. 6:**
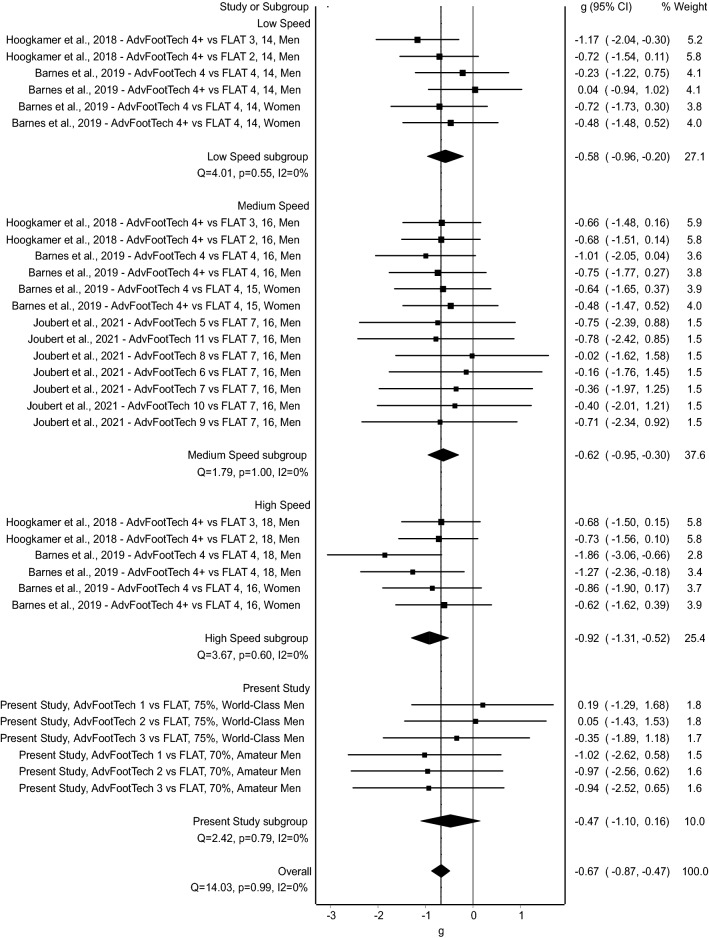
Forest plot displaying oxygen cost of transport (mL/kg/km) comparisons between advanced footwear technology (AdvFootTech) and traditional racing flats (FLAT) sub-categorized into different speeds. Study labels consist of the study name, the examined AdvFootTech versus FLAT condition where + indicates conditions that are weight matched, the speed either in km/h or as a % of peak, and the examined population. *CI* confidence interval

The meta-analysis of oxygen cost of transport (mL/kg/km) of the three studies that included this data revealed a statistically significant benefit of advanced footwear technology on the oxygen cost of transport measures [mean (95% CI); *g* = − 0.67 (− 0.87, − 0.47), *Z* = − 6.60 (*p* = < 0.001), Fig. 6]. Considering the subgroup analysis by speed, a medium effect [*g* = − 0.58 (− 0.96, − 0.20)] was found at low speeds, a medium effect [*g* = − 0.62 (− 0.95, − 0.30)] at medium speeds, and a large effect [*g* = − 0.92 (− 1.31, − 0.52)] at high speeds. Incorporating the data presented in this study, an overall medium effect [*g* = − 0.47 (− 1.10, 0.16)] was found. Here as well, no statistically significant heterogeneity was found (*Q* = 14.03, *p* = 0.99) and inconsistency was very low (*I*^2^ = 0%) among the examined studies [45].

**Fig. 7:**
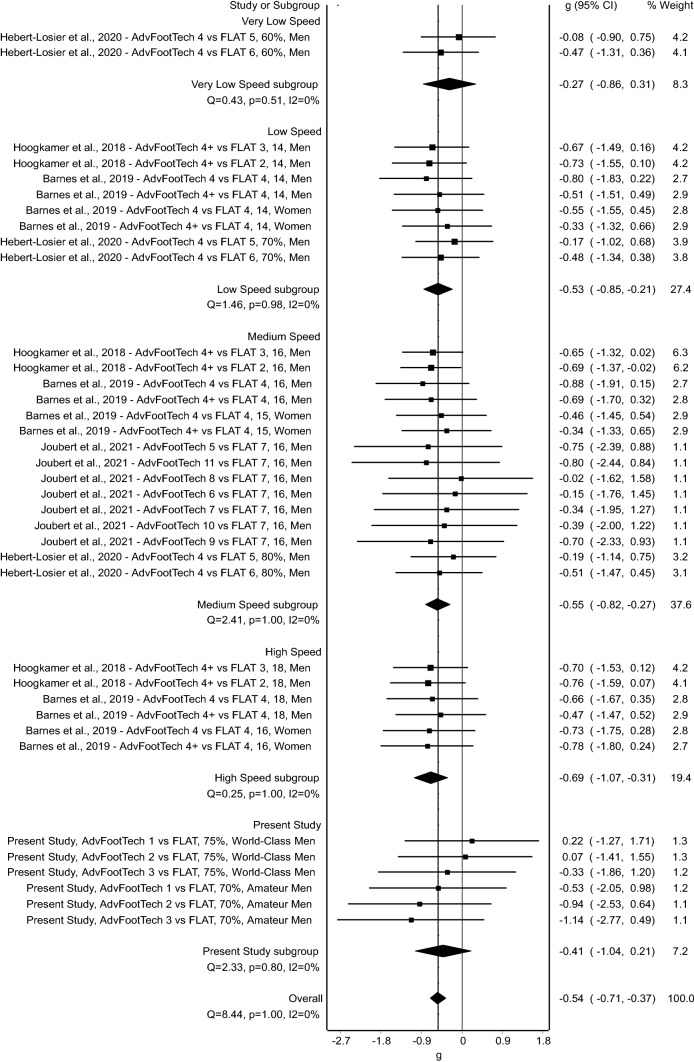
Forest plot displaying energetic cost (W/kg) comparisons between advanced footwear technology (AdvFootTech) and traditional racing flats (FLAT) sub-categorized into different speeds. Study labels consist of the study name, the examined AdvFootTech versus FLAT condition where + indicates conditions that are weight matched, the speed either in km/h or as a % of peak, and the examined population. *CI* confidence interval

Finally, the meta-analysis of energetic cost (W/kg) of the four studies showed a statistically significant benefit of advanced footwear technology on energetic cost measures [mean (95% CI); *g* = − 0.54 (− 0.71, − 0.37), *Z* = − 6.28 (*p* = < 0.001), Fig. 7]. Further examination of the subgroup speed analysis shows a small effect [*g* = − 0.27 (− 0.86, 0.31)] at very low speeds, a medium effect [*g* = − 0.53 (− 0.85, − 0.21)] at low speeds, a medium effect [*g* = − 0.55 (− 0.82, − 0.27)] at medium speeds, and a large effect [*g* = − 0.69 (− 1.07, − 0.31)] at high speeds. Analysis of the present study shows an overall medium effect [*g* = − 0.41 (− 1.04, 0.21)]. Again, here, no statistically significant heterogeneity was found (*Q* = 8.44, *p* = 1.00) and inconsistency was very low (*I*^2^ = 0%) between the subgroups [45].

## Discussion

In this study, we aimed to assess the variability in running economy in advanced footwear technology compared to a traditional racing flat on a treadmill in world-class Kenyan versus European amateur runners at speeds proportional to a marathon pace. Our laboratory results revealed ± 11.4% variability of the running economy of different advanced footwear technology running shoes in world-class Kenyan road runners, while for amateur Europeans, results range from a 9.7% benefit to a 1.1% drawback. The post-hoc meta-analysis revealed an overall statistically significant medium benefit of advanced footwear technology on running economy when compared with traditional flats.

### Running Economy and Running Performance Inter-Individual Variability

The running economy of the measured advanced footwear technology compared to a traditional racing flat of all tested subjects revealed a large inter-subject variability with overall values that ranged from an 11.4% benefit to an 11.3% drawback (Fig. 3). To compare this variation of running economy to other studies, we conducted a systematic literature search. Interestingly, this revealed similar variability in the found research considering the obtained confidence intervals in the conducted meta-analysis (Figs. 5, 6, 7). Hoogkamer et al. examined for the first time advanced footwear technology versus previously established marathon racing flats, all mass neutralized, in high-caliber athletes at three distinct speeds. The results found a range of 1.97–6.26% benefit in energetic cost (W/kg) of the new advanced footwear technology versus flats [28]. A similar study conducted by Barnes and Kilding showed a 1.72–7.15% running economy benefit (mL/kg/min) in highly trained runners in favor of the advanced footwear technology with only trivial-to-small differences between the tested men and women [15]. On average, this study found a 4.2% running economy benefit of advanced footwear technology versus the flat, which decreased to 2.9% when these conditions were weight matched, indicating the effect weight might have on such testing [15]. In an additional study, Hunter et al. found a response range of a 0.0–6.4% improvement in running economy (mL/kg/min) for advanced footwear technology and further suggested that different runners may require individualized shoe stiffnesses to enhance performance [29]. Hébert-Losier et al. examined both running economy and performance during a 3-km time trial and found a variability in running economy (mL/kg/min) of a worsening by a 10.3–13.3% improvement across conditions in recreational runners, and a time trial variability of a worsening by a 4.7–9.3% improvement [27]. To compare seven different models of advanced footwear technology, Joubert et al. conducted running economy tests (mL/kg/min) with trained distance runners and found that when all advanced footwear technology shoes are combined, the responses, as calculated from presented mean and standard deviations as well as described values, ranged from a 1% disadvantage to a 5.3% advantage [30]. An additional group of research studies also conducted a similar analysis by examining race performance measures instead of physiological data obtained in a laboratory. Considering these as well, Guinness et al. examined marathon race performance results from hundreds of elite marathoners who switched to advanced footwear technology and found that 74.5% of the men ran faster with an estimate of a 1.4–2.8% improvement in performance, while 71.4% of the women ran faster with an estimate of a 0.6–2.2% performance improvement [47]. Similarly, Senefeld et al. further examined performance and racing shoes in elite racers in four major marathons and found that in a subgroup of athletes with subsequent race performance of a flat then advanced footwear technology, the between-race change in performance for female athletes had a 95% confidence interval range from a 6.9% hindrance to a 13.8% advantage and a 5.4% hindrance to an 11.4% advantage in male athletes, suggesting that observed findings in a laboratory setting translate to real improvements in racing conditions [48]. Finally, Bermon et al. analyzed seasonal best times throughout the years to determine the effect of switching to advanced footwear technology, and found that in half-marathon and marathon races of a subgroup of athletes who competed in the same event with and without these shoes, all athletes (except male half-marathon runners) significantly improved their performance times with calculations on presented data showing that on average the female athletes showed a greater benefit of 1.9% faster in both races when compared with a 0.8% better performance found in the male athletes [49]. Overall, comparable to the present study, the variability in previously published data range from a 13.8% benefit to a 10.3% drawback in an overall change in performance of advanced footwear technology versus traditional racing flats as measured both in the laboratory with steady-state running physiology tests, and in the field examining race times.

Additional results from the five studies included after a retrospective systematic review and meta-analysis revealed that advanced footwear technology had an overall significant medium effect of − 0.58 when compared with a flat in terms of running economy, oxygen cost of transport, and energetic cost, even when accounting for the large individual variability found in these individual studies [15, 27–30]. Interestingly, as revealed via the subgroup analysis, the effect changed with the speed sub-groups where very low speeds showed a small effect and high speeds showed a greater effect, aligning with what has previously been shown in the literature [50]. This suggests that mechanisms involved in the advanced footwear technology might be proportional to the other biomechanical aspects such as changes in stride or gait cycle that alter with speed, with the mechanism reducing the energy required for running bouts proportionally higher when running at higher speeds [51].

Despite the findings of the meta-analysis, it remains important to consider the great inter-individual differences in the response to footwear conditions with individuals in the presented study as well as subjects in previous research showing significant inter-individual differences. Such results suggest possible methodological limitations of measuring the performance of running shoes (e.g., laboratory-based studies, insufficient familiarization protocols), as well as the importance of an individualized approach for athletes considering different biomechanical or anthropometrics that could be contributing to optimize their response to advanced footwear technology.

### Intra-Individual Running Economy Differences in Shoe Conditions

When examining the individual cases, some subjects showed meaningful effects depending on the specific advanced footwear technology shoe being tested, and others were not always trending the same way among all advanced footwear technology models. For example, given the results here, one of the world-class Kenyan runners showed a range from an 11.4% to a 0.2% benefit in the different advanced footwear technology models (Fig. 3A). For the aforementioned athlete, comparing personal best half-marathon times, this individual did indeed improve a sub-1-h half-marathon time by over 1:20 (min:s) in a shoe where this athlete was more economical during testing [52]. However, for another world-class subject who exhibited a running economy range of a 2.5% benefit to a 6.6% drawback for different advanced footwear technology, comparing marathon seasonal best times, this athlete was able to set a new personal record by reducing 2 min off a time already under 2:10 (h:min) in shoes that they, according to our test, should have performed worse in. This further affirms possible limitations of testing shoe performance in this way, particularly with a world-class Kenyan running population where further confounders such as a lack of familiarization to treadmill running and testing conditions might be playing a role.

### Populations Running Economy Differences

When examining in our study the differences in variability ranges between the world-class (an 11.4% benefit to a 11.3% drawback) and the amateur (a 9.7% benefit to a 1.1% drawback) populations, further exploration into the data revealed possible explanations. As we did not measure the running economy of all participants at the same speed, we are unable to conclude how the running efficiency of these two populations compared as a baseline in the same traditional racing flat. However, previously published research established that East Africans have a running economy advantage when compared with their Spanish counterparts [12]. Therefore, one consideration could be that our world-class cohort was already more economical when running in the traditional racing flat and therefore would not benefit as much when compared to the amateur European population.

Additionally, regarding the methodology, certain differences between the two populations are also apparent. First, while the relative effort between populations might be comparable, the speed at which they attained such effort differed with the average submaximal velocity for the world-class runners being 17.1 ± 0.4 km/h compared with 13.1 ± 1.0 km/h of the amateurs. These differences could be affecting the percentage benefits of advanced footwear technology in regard to running economy [53]. Moreover, even with a brief warm-up and familiarization session, some world-class runners were not used to running on a treadmill, which as Colino et al. has suggested, changes the mechanics compared with overground running [54, 55]. Furthermore, of note, at the point of testing, the world-class population had already been training in a version of the advanced footwear technology and were therefore familiar with the high-stack height and the feel of running with this technology. In contrast, the amateurs were not regularly running in such shoes outside of the present study. Previous research conducted has suggested injury risks and possible biomechanical changes when transitioning to novel footwear (e.g., minimalist shoes) too quickly, recommending a longer adaptation period [56–58]. Both considerations could have biased the results of the present study.

### Limitations

Several limitations to this study must also be acknowledged. First, we acknowledge the present study is underpowered. As no previous study had been conducted examining a world-class cohort, we had to do power and sample size calculations post-hoc. To start with the amateur cohort, using the smallest found effect size of 0.47 for running economy, sample size calculations revealed that 14 participants should be considered for such an analysis, consistent with the 14 total participants we had recruited at the start of the experiment. Using this same effect size for the amateur cohort, calculations revealed a power of 46.2%. When considering each cohort separately, as with most other studies examining sub-elite populations, we were able to see differences in advanced footwear technology for the amateurs. For the world-class cohort, the effect sizes for running economy of advanced footwear technology shoes compared to the flat varied from 0.04 to − 0.30. Considering this range in effect size, the power calculation here revealed a 5.2% up to a 20.4%. As this signifies our study as being underpowered, we also calculated the necessary sample size that would be needed for the world-class cohort to achieve the desired power of 80%. Based on which effect size, results here revealed 32–1705 participants would be needed, which is a challenge to maintain the high level required in such a large group of participants. This is a common issue that studies using world-class athletes are often underpowered given the singularity and inaccessibility to this sample, resulting rather in case studies or studies with a limited sample size [59]. With the world-class athletes, we must also consider the margin of the examined population, where even a minimal improvement in efficiency can reduce the finishing time over the duration of a marathon and could be the difference between a podium place or not. Furthermore, the results reflect that we must consider the large inter-subject variability and therefore the individuality of the athletes. The question remains of how to detect the marginal changes in an elite population. To further examine this, future studies should also consider examining the test–retest reliability of steady-state running economy laboratory assessments conducted on world-class athletes.

Additional limitations must also be considered owing to the athletes’ schedules and availability. More time would have also allowed us to repeat testing measures with the athletes, which would have ensured further reliability of the testing. An additional limitation was that no female athletes were tested within the scope of this study as we only had access to male athletes. Previous results considering both sexes range from only trivial to small differences in laboratory testing to significant differences in performance finishing times for female athletes [15, 48, 49]. Furthermore, it is important to note that because the intention was to test with shoes readily available on the market, it was impossible to blind the participants as to the shoe they were testing. As mentioned, because some athletes were already familiar with and training in versions of these shoes, athletes may have had pre-established opinions that could have influenced the results and the placebo effect cannot be excluded [29]. It must be noted, however, that related research comparing the running economy of different shoes where subjects were blinded to the shoes that were painted in black still revealed similar results [27].

Limitations related to the systematic review and meta-analysis include methodological and characterization variations. For example, some studies manipulated the shoe conditions in terms of weight matching or spray painting for blinding. Additionally, the ambiguity in subject definition related to the caliber of runners makes it difficult to place the results according to populations. Finally, with respect to the described shoe conditions, the specific model or version of a shoe within a franchise was not always clearly labeled, thus we had to make an informed categorization based on the information available.

## Conclusions

Next-generation long-distance running shoes that contain advanced footwear technology result in large inter- and intra-subject variability when measured for changes in running economy in both world-class Kenyan and amateur European runners with overall values that range from an 11.3% hindrance to an 11.4% benefit. Similar variability was also found in the literature as measured both in the laboratory and with real race performance. Additionally, meta-analysis results reveal an overall significant medium benefit of advanced footwear technology on running economy when compared with traditional flats. Such results have important indications. First of all, while testing the performance of shoes with running economy tests has become standard practice, further research should consider other methods that ensure ecological validity, which could include repeated economy tests or field-based tests. Furthermore, performance testing should be standardized to get a better comparison between studies. This is particularly important for the world-class athletes where additional constraints could be affecting their results as well as the acknowledgment that they may already have a better running economy. Second, this study acknowledges that a more personalized approach is necessary and that, when confirmed with additional testing, the inter- as well as intra-subject variability should be considered by stakeholders involved in elite sport. First, among others, it could affect athletes and coaches regarding their shoe selection; sport associations should acknowledge the importance of individualization in sport; shoe manufacturers should consider this when implementing new technology; and governing bodies should consider what impact this might have on the sport, with regard to which magnitude of effect is acceptable and fair.

## Declarations

### Data availability

Considering the inherent characteristics of this research, the participants of this study did not agree to publicly share the obtained individual data.
